# Statistical analysis of PML incidences of natalizumab-treated patients from 2009 to 2016: outcomes after introduction of the Stratify JCV® DxSelect™ antibody assay

**DOI:** 10.1007/s13365-016-0482-z

**Published:** 2016-10-11

**Authors:** Denise Campagnolo, Qunming Dong, Lily Lee, Pei-Ran Ho, Diogo Amarante, Harold Koendgen

**Affiliations:** Biogen, 14 Cambridge Center, Cambridge, MA 02142 USA

In January 2012, the presence of John Cunningham virus (JCV) antibodies was incorporated into the US prescribing information for Tysabri® (natalizumab) as a third independent risk factor for progressive multifocal leukoencephalopathy (PML) development in natalizumab-treated patients (Tysabri 2016). The assay used to detect the presence of JCV antibodies, Stratify JCV® DxSelect™ (Focus Diagnostics, Cypress, CA, USA), reports a binary output of positive or negative. Additional research described in 2013 (Plavina et al. 2013) and published in 2014 (Plavina et al. 2014) demonstrated that JCV index values could further stratify PML risk in natalizumab-treated anti-JCV antibody positive patients without prior exposure to immunosuppressants.

The estimated US incidence of PML risk table (Tysabri 2016) helps to inform health care providers (HCPs) and their patients about PML risk on natalizumab treatment. It is based on three identified risk factors: the presence of anti-JCV antibodies, the duration of treatment (especially beyond 2 years), and prior treatment with an immunosuppressant. Benefit/risk assessment is complex and varies among HCPs and patients. The algorithm exists to assist HCPs in risk stratification of PML based on the established risk factors. On the assumption that risk stratification has altered patient management, the outcome of potentially changing behavior can only be assessed retrospectively and after a significant period of time.

Dr. Werner has reported (Werner and Huang 2016) that in the nearly 4 years since the antibody test became available as a means of risk stratification, there has been only a modest reduction in the incidence of PML arising from natalizumab treatment (based on data from February 2011 to January 2016).

**Fig. 1:**
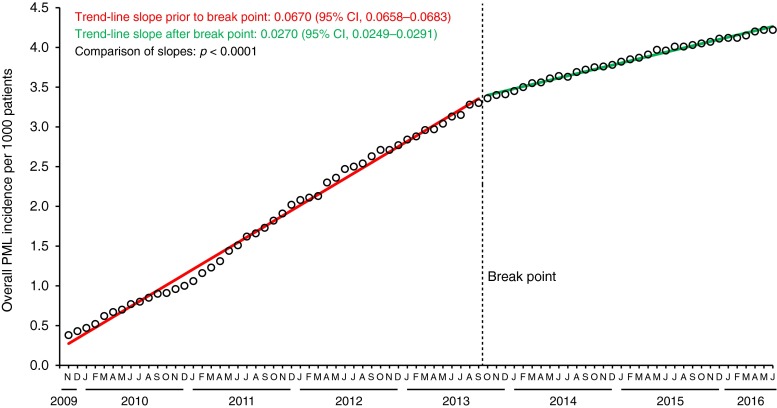
Incidence of PML in natalizumab-treated patients from 2009 to 2016. Piecewise regression analysis, incorporating changing points, was employed to identify the optimal break point and to estimate the slopes. Statistical testing shows a two-piece regression is significantly better than one-piece regression, whereas three-piece regression does not give a significantly better fit for the data than two-piece regression. *CI* confidence interval

Using a broader data set of PML incidence extending from November 2009 to June 2016 (Biogen, data on file), we generated a new graphical representation of PML incidence in natalizumab-treated patients (Fig. 1). While our analysis is similar to that of Dr. Werner, we also applied a statistical method to approximate the plotted curve, as described below. The two lines drawn in red and green were found to be the best-fit lines. (The statistical method demonstrated that the best fit is not substantially improved by approximating the curve with three or more lines.) The differences between the associated slopes are statistically significant (*p* < 0.0001). As shown on the graph, PML incidence increased at a rate of 0.067 per month from December 2009 to September 2013. From September 2013 to June 2016, the slope of the line increased at a rate of 0.027 per month, representing a more than 50 % decrease in the slope after September 2013. The mathematically derived break point, which can be tested at any point along the curve, was optimized at the September 2013 time point, approximately 18 months after introduction of the JCV antibody assay.

While it is not possible to assign causal relationships for the slope reduction, a significant reduction in PML incidence rise is observed beginning in September 2013 and continuing through June 2016. Further investigation may elucidate factors that have influenced this change over time in PML incidence.
